# Wheat oligopeptides enhance the intestinal mucosal barrier and alleviate inflammation via the TLR4/Myd88/MAPK signaling pathway in aged mice

**DOI:** 10.29219/fnr.v66.5690

**Published:** 2022-02-14

**Authors:** Yang Xian, Pan Da, Yang Chao, Xia Hui, Yang Ligang, Wang Shaokang, Sun Guiju

**Affiliations:** Key Laboratory of Environmental Medicine and Engineering, Ministry of Education/Department of Nutrition and Food Hygiene, School of Public Health, Southeast University, Nanjing, P.R. China

**Keywords:** aging, intestinal mucosal impairment, intestinal barrier, wheat oligopeptides, anti-inflammatory

## Abstract

**Background:**

Aging can induce oxidative stress, inflammation and mucosal impairment, and few works have been conducted to investigate the protective effects of WP on the natural intestinal aging process.

**Objective:**

The present work aimed to examine the protective effect of wheat oligopeptides (WP) on intestine mucosal impairment in aged mice, and investigate the potential antioxidation, anti-inflammatory effects of WP.

**Design:**

Seventy-two aged mice (24 months old) were randomly divided into six groups, 12 for each group. Twelve young mice (6 months old) were regarded as the young control group. WP (25, 50, 100, 200, or 400 mg/kg) or distilled water were administered daily by gavage for 30 days.

**Results:**

Histological observations showed that intestinal mucosal degeneration was attenuated by WP pretreatment. WP exhibited remarkable antioxidant activity via increasing superoxide dismutase, glutathione peroxidase, total antioxidant capacity and catalase activities, and decreasing the malondialdehyde levels in small intestine mucosa. WP pretreatment significantly suppressed intestinal mucosa inflammation through the reduction of TNF-α, TGF-β, IFN-γ IL-1β and IL-6. WP markedly protect the intestinal mucosal barrier by decreasing the ICAM-1 level, and increasing ZO-1 and JAMA-A levels. WP significantly down-regulated protein expression levels of TLR4, Myd88, and MAPK, suggesting that WP have a potential effect on inhibiting aging-induced inflammatory responses by blocking TLR4/Myd88/MAPK signal transduction.

**Conclusion:**

WP administration effectively alleviated intestinal mucosal impairment in aged mice. The potential mechanism was associated with enhancement of antioxidation and anti-inflammatory action and protection of the intestinal mucosal barrier.

## Popular scientific summary

- Wheat oligopeptides (WP) exhibit diverse biological activities, including anti-oxidative and anti-inflammatory activity.
- WP treatment caused significant reduction of TNF-α, IL-1β, IL-6, and IFN-γ levels in intestinal mucosa.
- WP markedly protect the intestinal mucosal barrier by decreasing the ICAM-1 level, and increasing ZO-1 and JAMA-A levels.
- WP have a potential effect on inhibiting aging-induced inflammatory responses by blocking TLR4/Myd88/MAPK signal transduction.

Aging is a process of progressive decline in the metabolic and physiological function of tissues and organs within the body with age and involves various diseases (1, 2). Aging organisms release inflammatory cytokines, reactive oxygen species, and growth factors which contribute to monolithic aging (3). The main characteristics of biological aging are oxidative stress, inflammation, and decreased physiological function (4), which provide the potentially mechanistic understanding of why the elderly people have an enhanced risk for chronic disease (5, 6). With the aged population expanding around the world, aging has become one of the most topical issue in the world (7). Therefore, it is extremely important to research and solve the problem of aging (8).

The function of the mucosal immune system is impaired in elderly people (9). Therefore, the incidence of gastrointestinal disease in older people is relatively high, which is an important cause of mortality. Weakened intestinal barrier and tissue inflammation are also major characteristics of the elderly (10). Therefore, it is extremely important to improve the fragile intestinal barrier and systemic inflammation. Currently, nutrition and exercise are considered as the probable non-genetic strategies to combat the harmful effects of gradual aging. Nutrition has been considered as the extremely reasonable and feasible way to alleviate the progression and severity of age and associated diseases and ‘nutrigerontology’ has recently been advocated (11). Some antioxidants have been verified to help in delaying senescence and preventing age-related diseases through mitigating oxidation (12, 13).

Wheat oligopeptides (WP) are a kind of bioactive oligopeptides obtained from wheat protein hydrolysate, which have many kinds of biological functions, including antioxidant (14, 15), anti-inflammation (16), antimicrobial (17, 18), and anticancer activities (19). A large number of researches have been conducted to identify and characterize these bioactive oligopeptides.

The technology of preparation and identification of WP has been well developed (15, 20, 21). The studies on WP mainly concentrated on the *in vitro* experiment, but in-depth researches on WP are rarely studied. Our previous studies indicated that WP can play a vital role in promoting growth on intestine epithelial cells (22), and exert protective effects against NSAID-triggered small intestinal injury in rats by reducing oxidative stress and modulating μ-opioid receptor (14).

The protective mechanisms underlying aging-mediated degeneration of intestine mainly include anti-oxidation and anti-inflammation. However, only few works have been conducted to investigate the protective effects of WP on the natural intestinal aging process and the potential mechanisms. The current study was applied to assess the effects of WP on oxidative and inflammatory pathways involved in naturally senile mice.

## Materials and methods

### Chemicals and reagents

WP were supplied by China National Research Institute of Food & Fermentation Industries (Beijing, China). The molecular weights of the WP were 140−1,000 Da and accounted for 92% of the total prepared WP and included a 3−6 amino acid sequence, prepared by hydrolysis of papain method. WP consist of 98.3% protein, 0.05% lipid, 4.56% ash content, and 4.21% water.

The TRIZOL Reagent Kit of superoxide dismutase (SOD), malondialdehyde (MDA), catalase (CAT), glutathione peroxidase (GSH-PX), epidermal growth factor (EGF), interferon-γ (IFN-γ), total antioxidant capacity (TAOC), Aminopeptidase N (APN), interleukin 1β (IL-1β), transforming growth factor-β (TGF-β), tumor necrosis factor-alpha(TNF-α), interleukin-6 (IL-6), zonula occluden (ZO-1), junctional adhesion molecule (JAM-A), and intercellular cell adhesion molecule-1 (ICAM-1) were purchased from Kiel biological technology Co.(Shanghai, China). Primary antibodies against TLR4, Myd88, p38MAPK, phosphor-p38MAPK, p44/42, and phosphor-p44/42 were purchased from Cell Signaling (Beverly, MA, USA). Horseradish peroxidase conjugated secondary antibodies and β-actin were purchased from Proteintech.

### Animals

Eighty-four male C57BL/6 mice (12 six-month-old mice in the young group and 72 24-month-old mice in the old group) were obtained from Vital River (Beijing, China) and housed under controlled environmental conditions of temperature (22±2°C), an entirely automated 12h/12h light/dark cycle. Mice were fed food pellets and given free access to drinking water. All animal experimental procedures were conducted in accordance with the guidelines of the Ethics Committee on the Care and Use of Laboratory Animals of Southeast University.

### Experimental design

After 1-week acclimation, 72 aged mice were divided into six groups (12 mice for each group): (1) aged control group; (2) 25 mg/kg WP; (3) 50 mg/kg WP; (4) 100 mg/kg WP; (5) 200 mg/kg WP; (6) 400 mg/kg WP. Twelve young mice were used as the young control group. The mice of young and old control groups were given vehicle (saline) and treatment groups were administered wheat oligopeptide daily for 30 days.

On the final day of the animal test, all mice were fasted for 24 h but were allowed free access to water. All mice were sacrificed under anesthesia, blood samples were obtained without addition of anticoagulants and then centrifuged for 10 min at 3,000*g* to obtain clear sera, which would be stored at −80 °C before use. After the mice were euthanized, the intestine was exposed and perfused with 10% buffered formalin after pylorus ligation and then fixed in 20 min.

### Histopathological observation

The small intestine samples removed from each mouse were fixed in 10% formalin solution for 24 h, dehydrated using graded alcohol and xylene, and embedded in paraffin. Paraffin sections were then cut into a thickness of 4 μm and stained with hematoxylin and eosin (H&E) for histological assessment. Sections were assessed by a blinded pathologist and scored for degeneration or necrosis of mucosal epithelial cells, transmural inflammation, the integrity of intestinal villi, infiltration of inflammatory cells in the lamina propria, and crypt abscessation on a scale from 0 (healthy) to 4 (most severe) (23). All tissue sections were evaluated under light microscope (Olympus BH2, Tokyo, Japan) at a magnification of 200×.

### Measurement of MDA level, CAT, GSH-PX and SOD activity

The levels of MDA and the activities of CAT, SOD and GSH-PX in intestinal mucosa were evaluated using commercial kits that had been purchased from the Nanjing Jiancheng Bioengineering Institute (Nanjing, China), following the manufacturer’s instructions.

### Measurement of TAOC and APN in intestine mucosa

TAOC and APN in intestine mucosa were determined using commercial kits that had been purchased from the Nanjing Jiancheng Bioengineering Institute (Nanjing, China), following the manufacturer’s instructions.

### Determination of IFN-γ, IL-1β, and EGF in serum

The levels of IFN-γ, IL-1β, and EGF in the serum were measured using an enzyme-linked immunosorbent assay ELISA kits (Kiel, China) for mice according to the manufacturer’s instructions.

### Determination of IL-6, ZO-1, JAM-A, TNF-α, TGF-β, and ICAM-1 in intestinal mucosa

Small intestine tissue samples were homogenized in 1 mL of phosphate buffer, followed by centrifugation of samples. The concentrations of IL-6, ZO-1, JAM-A, TNF-α, TGF-β, and ICAM-1 were detected using commercial enzyme-linked immunosorbent assay (ELISA) kits (Kiel, China) at a wavelength of 450 nm complying with the manufacturer’s instructions.

### Western blot analysis

Intestinal tissue samples were lysed in a RIPA lysis buffer and the protein concentration was determined using the BCA protein assay kit. Equivalent extracts were separated by 12% SDS-polyacrylamide gel electrophoresis and blotted onto a polyvinylidene difluoride membrane. The membranes were incubated with specific primary antibodies at 4°C overnight. Whereafter, the membranes were subjected to proper secondary antibodies incubation. The detection of the protein bands was carried out using enhanced chemiluminescence detection reagent. The densities of bands were measured using the Image J software.

### Statistical analysis

Data were represented as mean ± SEM of 12 mice per group. Differences between groups were analyzed statistically using one-way ANOVA followed by Dunnett’s test. SPSS statistical software version 22.0 was used to analyze the data according to the respective statistical techniques. Values were considered statistically significant when *P* < 0.05.

## Results

### WP Attenuated intestinal naturally aging in aged mice

**Fig. 1:**
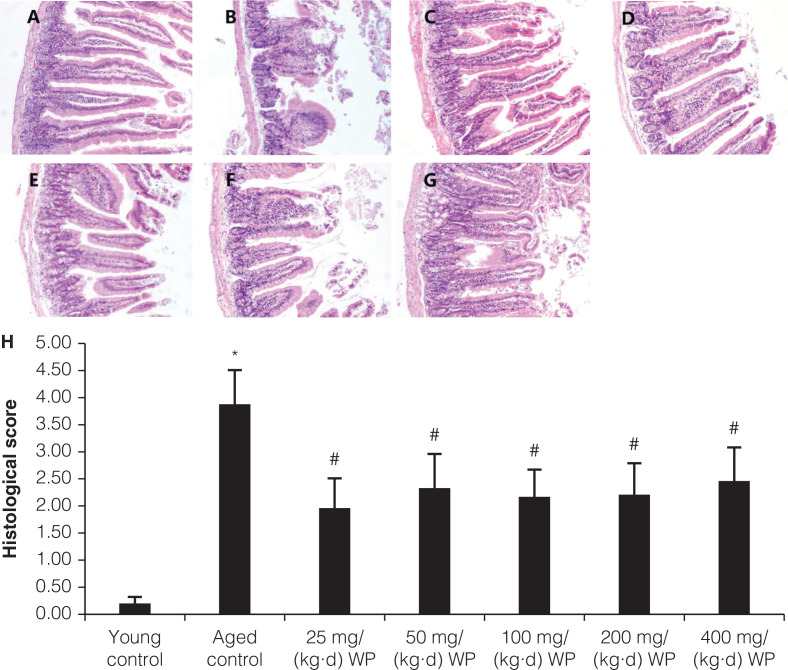
Histological evaluation of small intestine tissue. (a) young control group, (b) old control group, (c) 25 mg/kg WP, (d) 50 mg/kg WP, (e) 100 mg/kg WP, (f) 200 mg/kg WP, (g) 400 mg/kg WP, (h) histological score.

As shown in Fig. 1a, in the young group, almost no intestine mucosal damage was detected. Villus height of the small intestine mucosa of the young control group is relatively consistent, and the structure of the villus is complete. The morphology of mucosal epithelial cells is normal and the nuclear membrane is clear. In the aged control group, the small intestinal mucosa villus (Fig. 1b) had local defects, became shorter and wider, and the villus density decreased. The mucosal epithelial cells became degenerated, necrotic, and exfoliated, and the infiltration of focal inflammatory cells in the lamina propria increased. Intervention of wheat oligopeptide can significantly improve the morphology of the small intestine, reduce the degeneration of epithelial cells, and decrease the infiltration of inflammatory cells and the density of villus (showed in Fig. 1c–h). The 25 and 50 mg/kg WP showed better protective effects.

### Changes of oxidative stress markers (MDA, CAT, GSH-PX, and SOD) in intestinal tissue

**Fig. 2:**
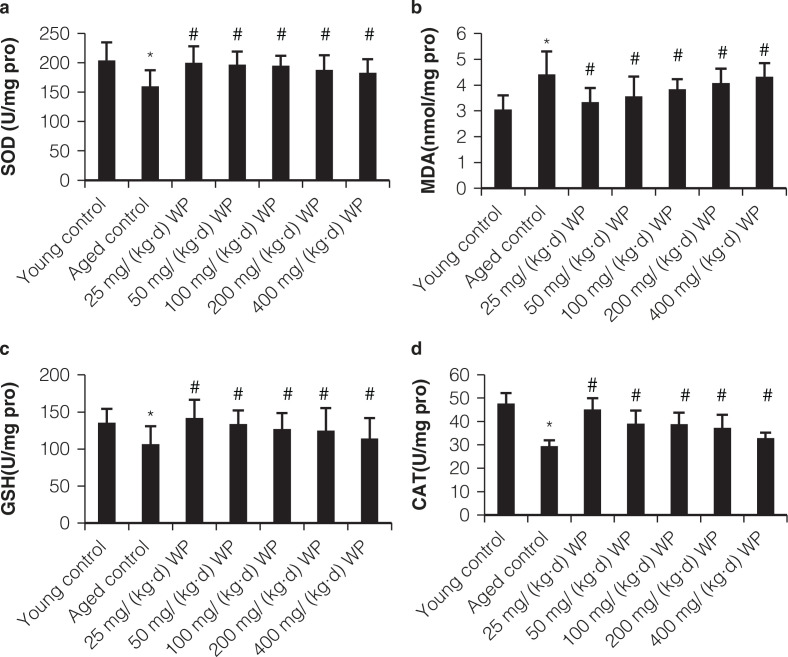
Effect of WP pretreatment on activities of enzymatic antioxidants such as superoxide dismutase (SOD), catalase (CAT), and Glutathione peroxidase (GPx) and levels of malondialdehyde (MDA) of intestine mucosa in mice. All values are expressed as mean ± SD (*n* = 12), * *P* < 0.05 significant versus young group, # *P* < 0.05 significant versus aging group.

As shown in Fig. 2, the activities of SOD, GSH-PX, and CAT significantly decreased, and the MDA level in the old control group significantly increased compared to the young control group (all *P* < 0.05). Moreover, WP treatment obviously enhanced SOD, GSH-PX, and CAT activities of the intestine tissue, as compared with the old control group (all *P* < 0.05). WP consumption significantly decreased MDA levels in intestine tissue when compared with the levels in the old control group. Convincing epidemiological evidence indicated that aging can significantly decrease the SOD, CAT, and GSH-PX activities and increase MDA levels.

### Changes of T-AOC and APN in intestine mucosa

**Fig. 3:**
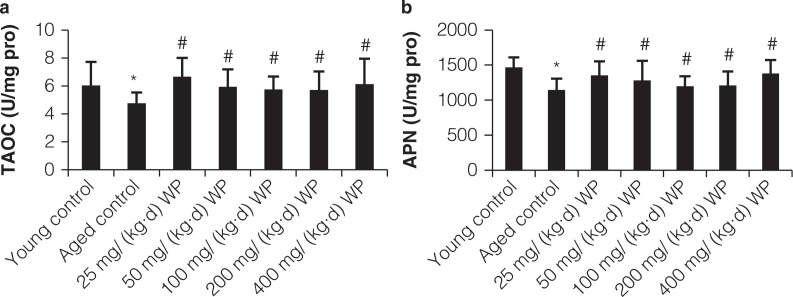
Effect of WP pretreatment on TAOC and APN of intestine mucosa in mice. All values are expressed as mean ± SD (*n* = 12), * *P* < 0.05 significant versus young group, # *P* < 0.05 significant versus aging group.

As shown in Fig. 3, the T-AOC levels reflect the non-enzymatic antioxidant activity of the defense system. Herein, the activity of T-AOC in intestinal mucosa of the old control group was markedly lower (*P* < 0.05) than that of the young control group. Moreover, compared with the old model group, the WP-treated groups showed an increase in the T-AOC levels in intestinal mucosa (*P* < 0.05, Fig. 3a). The activity of APN in intestinal mucosa of the old mice was obviously lower (*P* < 0.05) than that of the young mice. Moreover, compared with the old model group, treatment of WP significantly increased the APN activity in intestinal mucosa.

### Changes of IFN-γ, IL-1β, and EGF levels in serum

**Fig. 4:**
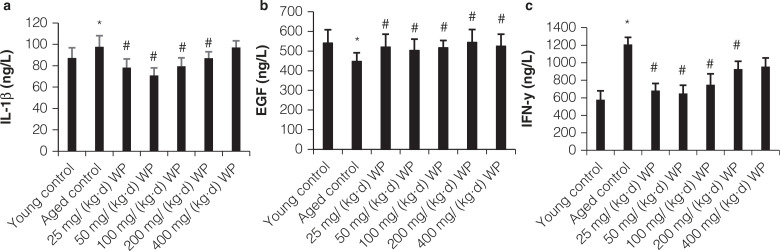
Effect of WP pretreatment on IL-1β, EGF, and IFN-γ content in serum of mice. All values are expressed as mean ± SD (*n* = 12), * *P* < 0.05 significant versus young group, # *P* < 0.05 significant versus aging group.

As shown in Fig. 4, the aged model group had higher levels of IL-1β relative to the control group (*P* < 0.05), and WP treatment significantly reduced the IL-1β level. Aging progress obviously augmented IFN-γ level, which could be suppressed by WP intervention. The level of EGF in serum decreased in the aging group in comparison to normal group, and pretreatment with WP exhibited an obvious increase of EGF level.

### Changes of JAMA-A, IL-6, TGF-β, ICAM-1, ZO-1, and TNF-α levels in intestinal mucosa

**Fig. 5:**
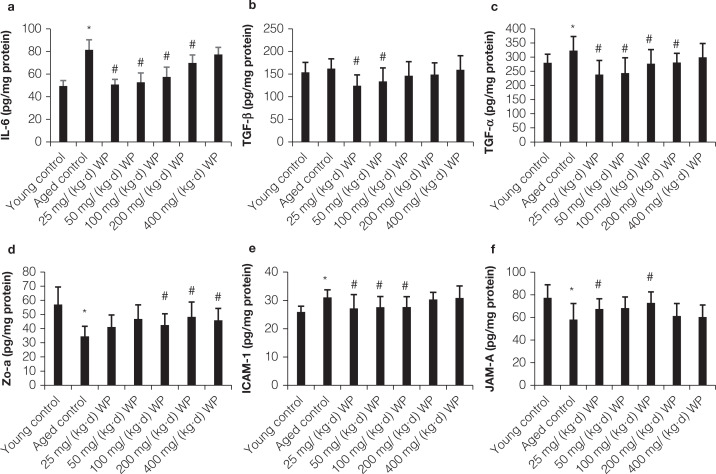
Effect of WP pretreatment on JAMA-A, IL-6, TGF-β, ICAM-1, ZO-1, and TNF-α levels in intestine mucosa of mice. All values are expressed as mean ± SD (*n* = 10), * *P* < 0.05 significant versus young group, # *P* < 0.05 significant versus aging group.

As shown in Fig. 5, the intestinal mucosa’ TNF-α level was significantly enhanced in old mice and WP pretreatment suppressed the increased TNF-α level in comparison with the old mice (*P* < 0.05). The levels of JAMA-A and ZO-1 in the aged model group were obviously decreased when compared with the young group. WP consumption significantly increased JAMA-A and ZO-1 levels in intestine mucosal. In addition, the intestine mucosal IL-6 level was significantly enhanced in the old group, and was significantly suppressed by pretreatment with WP (*P* < 0.05).

The ICAM-1 levels in intestine tissue were markedly increased in the aging mice in comparison to the normal group (*P* < 0.05). WP administration indicated a considerable decrease in the levels of TGF-β and ICAM-1 in intestine tissue.

### WP targets intestinal TLR4, Myd88, and MAPKs pathway

**Fig. 6:**
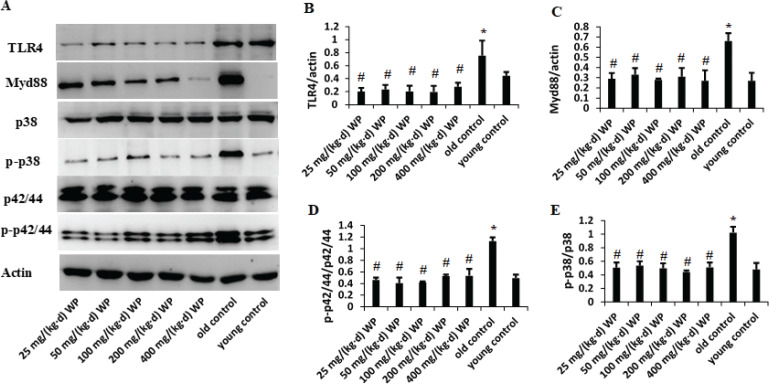
Effect of WP pretreatment on expressions of the inflammatory marker TLR4, Myd88, and MAPKs in intestinal tissue. Intestine homogenates were used for the analysis of protein expression of TLR4, Myd88, and MAPKs.

The signaling pathway of MAPKs in intestinal tissue was studied to further explore the inflammatory conditions. Western blot analysis of intestinal tissue showed that the aging progress significantly increased expression of TLR4 and Myd88 in comparison with the normal group (Fig. 6a–c). Treatment of mice with WP significantly prevented the expression of TLR4 and Myd88. Taken together, these results indicated that WP suppresses inflammatory response through downregulating the expression of TLR4 and Myd88.

According to previous research, MAPKs pathways may affect the release of proinflammatory cytokine and transduce TLR4 signals. The present study investigated the effect of WP consumption on aging mediated activation of p38 and p42/44. As shown in Fig. 6, p38 and p42/44 phosphorylation in the intestine tissues of aged mice were markedly induced. WP pretreatment obviously suppressed aging-mediated phosphorylation of p38 and p42/44.

## Discussion

WP exhibit diverse biological activities, including antioxidative and anti-inflammatory activity. The current study highlights the protective effect of WP against natural degeneration of the small intestine in aging mice at five doses (25, 50, 100, 200, and 400 mg/kg). The results indicate that aging can lead to oxidative stress, inflammation, and degradation of intestinal morphology and function, which is similar to previous studies (9, 24). The results also suggest that WP confer considerable protection to the mice intestinal mucosa degeneration via anti-inflammatory and anti-oxidative mechanisms.

Five doses of WP were used in the naturally aging mice model to assess an optimal dose. In our present study, the 25 and 50 mg/kg WP intervention exerted optimal protective effect by maintaining oxidative stress, inflammatory response, and intestinal barrier near young control. The higher doses of WP tested also exert dramatic protection from intestinal degeneration, but to a lesser degree. This negative dosage–effect relationship has been reported previously (14), however, more researches need to be conducted to confirm whether the lower dosage is the optimal dosage, and the lowest effective dose. This research displays that WP is effective in protecting aging-mediated intestinal impairment.

Aging can induce oxidative stress and impair antioxidant defense in experimental animals. Oxidative stress may lead to physiological dysfunction through the imbalance of the antioxidant/prooxidant ratio. The pathogenesis of aging may include production of oxygen derived free radicals, mainly hydroxyl radicals, superoxide anions, and lipid peroxides (25). Lipid peroxidation and free radical-induced antioxidizing enzymes inhibition may be conducive to intestinal mucosal degeneration (26). Antioxidant enzymes such as SOD, CAT, and GSH-Px play an important role in arresting the formation of reactive oxygen species and protecting the cells from the impairments induced by free radicals (27). As a secondary product of lipid peroxidation, accumulation of MDA is often used as an indicator to quantify and identify lipid peroxidation (28). As shown in Fig. 2, the highest contents of MDA but lowest activities of SOD, GSH-Px, as well as T-AOC were found in the aging group.

Our results support the fact that oxidative stress plays a vital role in the pathogenesis of aging intestine. Nevertheless, pretreatment with WP led to marked increases in the activities of SOD, GSH-Px, CAT, and T-AOC as well as a reduction in MDA formation, demonstrating its antioxidant activity. The present results suggested that WP potentially exhibited protective effect on aging intestine through antioxidant mechanism.

Aminopeptidase plays a vital role in the process of protein digestion and is widely distributed on the brush border of small intestinal mucosal epithelial cells (29). Most oligopeptides can only be absorbed after being hydrolyzed or transported into the cells by cytoplasmic peptidase in the brush border of the epithelial cells of the small intestine (30). The results showed that the activity of APN in the small intestinal mucosa of the elderly group was significantly reduced, when compared with the young group. WP treatment markedly increased the APN activity.

Epidermal growth factor (EGF) could accelerate protection and restoration of intestinal mucosa, primarily through activating Na+/H+ exchange of epithelial cells (31). Enhancement of EGF in intestinal mucosa can stimulate proliferation and migration of epithelial cells and accelerate epithelial regeneration and repair process (32). The treatment with WP caused a marked increase in EGF levels in serum. The present study revealed that the protective effects of WP on intestinal mucosa occurred by promoting the secretion of EGF.

Inflammation has been increasingly regarded as an important pathophysiological phenomenon in ageing (33). The present results indicated that aging significantly augmented inflammatory mediators, including TNF-α, IL-1β, IL-6, and IFN-γ, which is similar with previous studies (34, 35). Because chronic inflammation is a risk for impairment of the intestine mucosal immune system and morphology, agents that downregulate inflammatory reaction may have therapeutic benefits for mitigating intestinal mucosal damage (9). The treatment with WP caused significant reduction in TNF-α, IL-1β, IL-6, and IFN-γ levels, implying that the WP was capable of relieving intestinal mucosal inflammation.

The authors further evaluated the protein expression levels of TLR4, Myd88, and MAPK signal transduction pathway. Recently, some studies demonstrated TLR4 were significantly increased in the healthy aging tissues (36), and involved in the downstream activation of MAPK (37, 38). TLR4 are found to be potential inducers of MAPK transcriptional activities (39). The disorders of the MAPKs pathway have been formerly described in intestinal pathologies, and present findings revealed that aging triggered an increased phosphorylation of p-p38MAPK, p-ERK1/2 levels. These findings are consistent with previous reports. These results indicated that WP pretreatment could mitigate intestinal impairment triggered by aging through the MAPK pathway. Furthermore, activation of ERK1/2 signal transduction is associated with the increase of epithelial proliferation and mucosal repair in naturally aging models.

Aging also affects properties of the intestinal barrier, possibly impacting on age-related local and systemic disturbances (40). Some pro-inflammatory cytokines such as IL-1β, IL-6, IFN-γ, and TNF-α may also influence mucosal barrier integrity and tight junction status (24). Inflammation stimulation can trigger up-regulation of ICAM-1, which enhanced intestinal epithelial permeability via triggering downregulation of myosin light-chain kinase and accelerating ligation of other apically localized proteins (41, 42). The present study indicated that the progress of aging induces upregulation of ICAM-1 in intestinal mucosa, whereas WP treatment significantly suppressed the ICAM-1 level.

JAM-A plays a direct role in the regulation of epithelial permeability and the mucosal inflammatory response (43). JAM-A can affect several cellular processes, including polarity, adhesion, and migration of cell (44). JAM-A deficiency results in increased colonic inflammation and paracellular permeability. Aging markedly decreased the JAM-A level in intestinal mucosa, and pretreatment of WP significantly increased the level of JAM-A when compared with aged mice. ZO-1 can also influence endothelial and epithelial permeability, and conserve barrier function (45). The lower level of ZO-1 in intestinal mucosa was observed in aged mice, and WP administration significantly increased the ZO-1 level in comparison to the aged group.

**Fig. 7:**
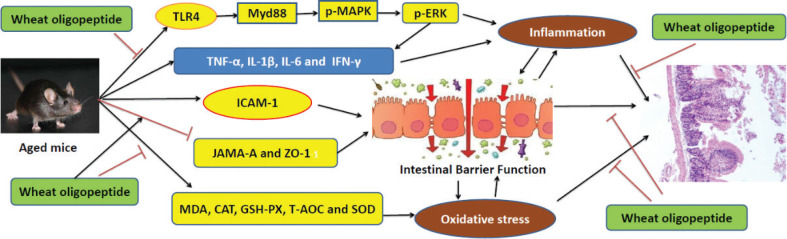
The mechanism pathways that intervened intestinal mucosal injury induced by aging and the intestinal protective effects of wheat oligopeptide. (→: activate; ┤: inhibit).

The present results indicated that wheat oligopeptide can markedly protect the intestinal mucosal barrier of aging mice by decreasing the intestinal mucosal permeability and preventing the intestinal bacteria from translocating (Fig. 7).

## Conclusions

Pre-treatment with WP displays an effective protection against aging-mediated intestinal mucosal oxidative stress and inflammation. WP can further promote repair of intestinal mucosa and maintain the intestinal mucosal barrier. These finding show a new perspective on natural aging and provide nutritional strategies to curb the progression as well as deleterious aspects of aging. Therefore, WP will be worthy of research to further confirm anti-aging effects and alleviation of age-related diseases, due to its ability to alleviate oxidative stress, inflammation response, and cellular senescence. In particular, the results suggested that wheat oligopeptide is likely to be a promising functional to curb the progression and cause an impairment of aging.
